# Changes in bone metabolic profile associated with pregnancy or lactation

**DOI:** 10.1038/s41598-019-43049-1

**Published:** 2019-05-13

**Authors:** Takeshi Miyamoto, Kei Miyakoshi, Yuiko Sato, Yoshifumi Kasuga, Satoru Ikenoue, Kana Miyamoto, Yuji Nishiwaki, Mamoru Tanaka, Masaya Nakamura, Morio Matsumoto

**Affiliations:** 1 Department of Orthopedic Surgery, Keio University School of Medicine, 35 Shinano-machi, Shinjuku-ku, Tokyo 160-8582 Japan; 2 Department of Advanced Therapy for Musculoskeletal Disorders, Keio University School of Medicine, 35 Shinano-machi, Shinjuku-ku, Tokyo 160-8582 Japan; 3 Department of Obstetrics and Gynecology, Keio University School of Medicine, 35 Shinano-machi, Shinjuku-ku, Tokyo 160-8582 Japan; 4 Department of Environmental and Occupational Health, School of Medicine, Toho University, 5-21-16 Omori-nishi, Ota-ku, Tokyo 143-8540 Japan; 5 Department of Orthopaedic Surgery, Faculty of Life Sciences, Kumamoto University, Kumamoto, Japan

**Keywords:** Osteoporosis, Prognostic markers

## Abstract

Calcium and nutrients are transferred from mothers to fetuses or infants during pregnancy or lactation, respectively, promoting metabolic changes in the mother, many uncharacterized. To evaluate these changes, we undertook two parallel studies. In one we analyzed fourteen clinical cases of vertebral fragility fractures, at or before three months after partum, in mothers who breastfed their infants. In the other, we enrolled 79 additional pregnant subjects, some who chose to breastfeed and others who did not, and analyzed changes in bone metabolic status starting between 34 and 36 weeks of gestation and ending one month after partum. In the larger group, bone-resorbing and bone-forming parameters such as serum TRACP5b and osteocalcin, respectively, significantly increased after partum. Among parameters that changed after partum, serum PTH and the bone-resorbing markers serum TRACP5b and urine NTX were significantly higher in mothers who only breastfed infants compared to mothers who fed infants formula or a mix of both. However, bone-forming parameters were comparable between breastfeeding and non-breast-feeding groups after partum, suggesting that elevated bone-resorption occurs only in the breastfeeding group. Radiographic analysis after partum demonstrated that no subject among the 79 analyzed showed vertebral fractures, even those who breastfed exclusively. Among fracture cases analyzed, subjects exhibited significantly lower bone mineral density than did non-fracture cases in breastfeeding-only subjects. We conclude that bone metabolic status significantly changes over the period between pregnancy and post-partum lactation, and that low bone mineral density seen in a small subset of breastfeeding-only cases likely causes post-partum vertebral fragility fractures.

## Introduction

Pregnancy and lactation alter skeletal metabolism in females^1^. During pregnancy, nutrients, in particular calcium, are supplied to fetuses from mothers, and after birth, lactation continues to supply calcium to infants. Thus, mothers must undergo increased calcium resorption from the intestine to satisfy infants’ needs, and osteoclastic bone resorption as well as osteocyteic osteolysis are activated to enhance calcium supply^1–5^, resulting in decreased bone mass in women^6^. Case reports have described fragility fractures in maternal skeletons after partum^1,7–11^. These conditions are infrequent and pathophysiological mechanisms underlying them are unknown.

During pregnancy and lactation, hormonal changes can perturb skeletal homeostasis. Serum levels of parathyroid hormone (PTH) or parathyroid-related protein (PTHrP) reportedly increase during lactation^1^. Elevated PTH levels promote osteoclast differentiation and activation, followed by increases in serum calcium levels due to activated osteoclastic bone-resorption, which in turn, reduces bone mass^12,13^.

Women show well-documented declines in bone mass after menopause^14^. Estrogen deficiency due to the menopause activates osteoclastic bone resorption, leading to osteoporosis, which is frequently associated with bone fragility fractures^15^. Similarly, after partum, mothers are in a transient condition of “menopause”, which likely alters bone metabolism. Osteoclasts reportedly express the estrogen receptor (ER), and either loss of estrogen or lack of ER in osteoclasts activates bone resorption in these cells^16,17^. These changes may occur not only in menopausal but in pregnant or breastfeeding women.

Bone homeostasis is regulated by integrated activity of bone-resorbing osteoclasts and bone-forming osteoblasts^18^, a process termed “coupling”^18^. Increased bone-resorption activates bone-formation, and if unregulated leads to reduced bone mass frequently seen in skeletal disorders such as postmenopausal osteoporosis^18,19^. The receptor activator of nuclear factor kappa B ligand (RANKL) plays a pivotal role in osteoclast differentiation and activation^20^, and lack of either RANKL or its receptor RANK completely abrogates osteoclastogenesis^21,22^. Interestingly, the RANKL-RANK system is also required for mammary gland development during lactation^23^, and female mice lacking either RANKL or RANK can become pregnant but cannot lactate. Thus, osteoclast formation and lactation are also considered coupled.

## Materials and Methods

### Subjects

Fracture subjects were female post-partum vertebral fracture patients aged 31–44 years at the first visit, who had been referred to our hospital with fragility fracture(s) of unknown origin between February 2014 and July 2017.

A different group of subjects examined for bone turnover markers before and after partum were pregnant females who had visited our hospital to give birth between February 2014 and April 2017. A total of 86 subjects were invited to this study, written informed consent was obtained from all individual participants, and each completed a self-reported questionnaire regarding past history and drug usage. Both fracture and non-fracture participants were asked at their first visit to the hospital and at the first visit after partum, respectively, to describe the way they fed their infants, such as through breastfeeding exclusively or use of formula, or mix of breast and artificial milk. All fracture subjects continued to breastfeed exclusively until they experienced fractures. Seven subjects were excluded due to refusal to undergo a follow-up examination, moving to another hospital or perinatal death, leaving 79 subjects enrolled. All subjects were Japanese women living in the Tokyo area. Both studies were approved by an ethics committee at Keio University School of Medicine and were carried out in accordance with clinical study guidelines.

### Measurements

Height, body weight, and body mass index (BMI) calculated from body weight and height data were assessed in all subjects. Sera and urine samples were collected from all subjects between 34 and 36 weeks of gestation and at one month after partum. Serum calcium (Ca), inorganic phosphorus (IP), creatinine, albumin, parathyroid hormone (PTH), TRACP5b and estradiol (E2) levels were assessed in all subjects. TRACP5b (Nittobo, Fukushima, Japan) and uDPD (Quidel Corporation, San Diego, CA) were analyzed by enzyme immunoassay (EIA). PTH (Roche diagnostics, Tokyo, Japan), osteocalcin (Roche), P1NP (Roche), E2 (Roche), prolactin (Roche) and ucOC (Sekisui Medical, Tokyo, Japan) were analyzed by electrochemiluminescent immunoassay (ECLIA). BAP (Beckman Coulter, Pasadena, CA) and uNTX (Alere Medical Co., Ltd, Tokyo, Japan) were analyzed by chemiluminescent enzyme immunoassay (CLEIA) and ELISA, respectively. 25(OH)D (DIAsource, Neuve, Belgium) was analyzed by radioimmunoassay. Bone metabolic parameters were examined at the first visit in fracture patients, and one month before and one month after birth in the group of pregnant subjects. Bone mineral density (BMD) was analyzed using a dual-energy X-ray absorptiometry (DEXA; GE Healthcare, Amersham Place, Little Chalfont, Buckinghamshire HP7 9NA, England) at the first visit in fracture patients, and one month after birth in pregnant subjects. Statistical analysis was undertaken using the unpaired two-tailed Student’s or Welch’s *t*-test (*p < 0.05; **p < 0.01; ***p < 0.001; NS, not significant). All data are shown as means ± S.D.

## Results

### Case reports of clinical vertebral fractures after partum

First, we evaluated 14 women showing clinical vertebral fragility fractures that occurred within three months after childbirth. None of these subjects had prior fracture histories before pregnancy. All patients were diagnosed with vertebral fractures at nearby hospitals and then referred to our hospital, where they underwent biological and radiographic examination. Three were excluded from the study, since they had been administered treatment for osteoporosis at least one year before visiting our hospital. The remaining 11 were on average 35.1 (31–44) years old at the first visit to our out-patient department (Table 1). Each had 1–8 (average 3.7) vertebral fragility fractures (Table 1), and all experienced severe back pain within three months after partum (Table 1) in the absence of traumatic accidents. All patients fed their infants breast milk exclusively. One patient took 10 mg prednisolone daily due to scleroderma and polymyositis, but the remaining subjects were free from disease and medications that might alter bone metabolic status. Osteoporotic vertebral fractures most often occur at the thoracolumbar junction; however, fracture sites seen in these patients after partum varied (Table 1). No cervical fractures were observed. Subjects exhibited bone mineral density (BMD) lower than −1.0 SD in lumbar spine. These results suggest that vertebral fragility fractures seen in these patients are likely due to low bone mass associated with metabolic changes associated with pregnancy and lactation.

**Table 1:** Characteristics of vertebral fracture patients after partum.

Case #	Age (years old)^*a^	# of vertebral fracture(s)	Fracture site	BMI (kg/m^2^)	LS BMD (T score)	Lt FN BMD (T score)	Rt FN BMD (T score)	Timing of onset (month after partum)	# of Baby	Note^*b^
1	32	1	L2	22.8	−2.6	−2.3	−2.3	1	1	
2	34	5	T11, T12, L1, L2, L3	18.3	−3.6	−2.6	−2.8	3	1	ALN
3	44	6	T10, T11, T12, L1, L3, L4	16.9	−3.2	−2.5	−2.3	2	2	
4	35	4	L1, L2, L4, L5	22.3	−1.3	−2.1	−1.9	3	1	
5	34	2	T11, L1	21.4	−1.8	−0.5	−0.2	2	1	MIN
6	33	3	T7, L1, L2	19.0	−2.9	−0.9	−1.1	2	1	
7	33	4	T9, T11, T12, L4	18.0	−3.0	−2.8	−3.1	1	2	PSL 10 mg/day
8	31	8	T6, T8, T9, T11, L2, L3, L4, L5	19.1	−1.9	−2.4	−2.4	2	1	
9	37	3	T11, L1, L511	16.6	−4.5	−3.3	−3.0	2	1	
10	36	2	T8, L2	17.4	−2.6	−1.4	−1.4	1	1	
11	37	3	T8, T11, L2	21.2	−1.3	−0.9	−1.2	2	1	
Average	35.1	3.7		19.4	−2.6	−2.0	−2.0	1.9	1.2	

^*a^At the first visit.

^*b^ALN, alendronate; MIN, minodronate; PSL, prednisolone.

### Changes in serum markers in non-fracture women before and after partum

Next, we analyzed changes in metabolic status in a different group of pregnant women (See Methods) during the period before and after partum. To do so, we invited 86 pregnant women who had visited our hospital to give birth. Informed consent was obtained from all, but 7 dropped out due to the requirement for a follow-up examination, relocation or death of their infant. The remaining 79 were enrolled in our study, and we took serum and urine samples from each subject between 34 and 36 weeks of gestation and after partum. We also undertook radiographic analysis including X-ray images of whole spine and dual energy X-ray absorptiometry one month after partum.

**Figure 1:**
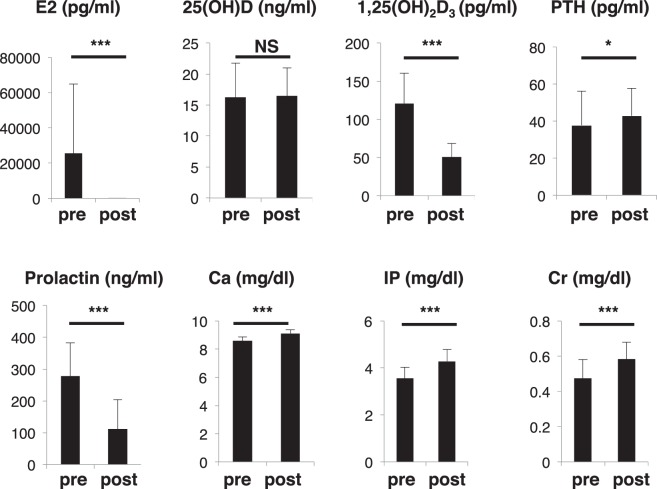
Changes in bone-related hormones and factors after childbirth. Sera were collected from a group of 79 pregnant subjects one month before (pre) and after (post) birth, and indicated parameters were analyzed and compared between groups. Data represent mean levels of indicated parameters ± SD (*n* = 79; *p < 0.05, ***p < 0.001, NS not significant). E2, estradiol; PTH, parathyroid hormone; IP, inorganic phosphorus; Cr, creatinine.

**Figure 2:**
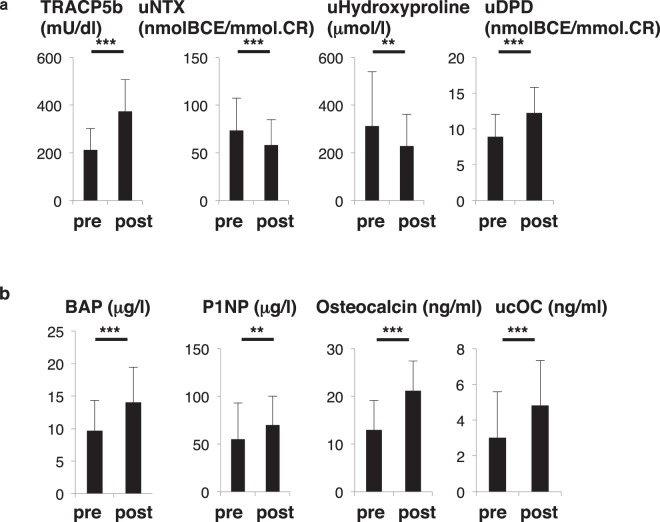
Changes in bone-resorbing and bone-forming parameters before and after birth. Sera and urine were corrected from pregnant subjects in the group of 79 one month before (pre) and after (post) birth, and levels of indicated parameters were analyzed (**a**, bone-resorbing parameters; **b**, bone-forming parameters) and compared between groups. Data represent mean levels of indicated parameters ± SD (*n* = 79; **p < 0.01, ***p < 0.001.). TRACP5b, tartrate resistant acid phosphatase isoform 5b; uNTX, urinary type 1 collagen cross-linked N-telopeptide; uDPD, urinary deoxypyridinolin; BAP, bone alkaline phosphatase; P1NP, procollagen type 1 amino-terminal propeptide; ucOC, undercarboxylated osteocalcin.

**Figure 3:**
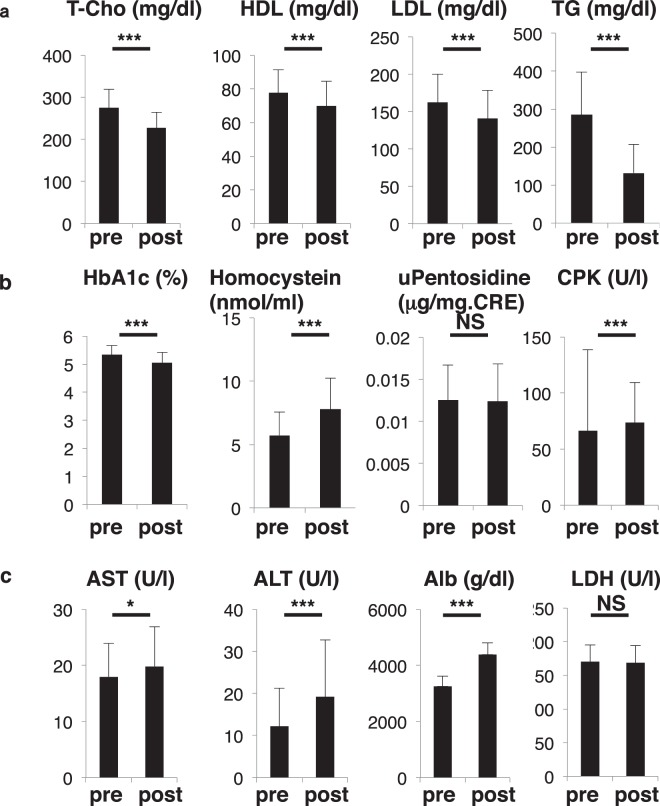
Metabolic parameters significantly changed following childbirth. Sera and urine were corrected from pregnant subjects in the group of 79 one month before (pre) and after (post) birth and analyzed for levels of indicated parameters (**a**, lipid parameters; **b**, glyco parameters; **c**, liver parameters). Levels were compared between groups. Data represent mean levels of indicated parameters ± SD (*n* = 79; *p < 0.05, ***p < 0.001, NS not significant). Pentosidine levels were analyzed in urine, and others in sera. T-Cho, total cholesterol; HDL, high density cholesterol; LDL, low density cholesterol; TG, triglyceride; HbA1c, hemogloblin A1c; CPK, creatine phosphokinase; AST, aspartate aminotransferase; ALT, alanine aminotransferase; Alb, albumin; LDH, lactate dehydrogenase.

Table 2 summarizes basic characteristics such as age, BMI before pregnancy, and number of other children at the post-partum stage of this study. No subject had a history of disease or medication use that might affect metabolic status. We then analyzed and compared serum and urine components in the 79 subjects before and after partum (Figs 1–3). We initially found that estradiol (E2) levels significantly decreased after partum, with an average 25,457 pg/ml pre-partum to 22 pg/ml post-partum, an approximately thousand-fold decrease (Fig. 1). This decrease was much more significant than that seen in pre- versus post-menopausal women, which normally go from ~174 pg/ml to <10 pg/ml^24^. This transient or temporary “menopause” after childbirth suggests that bone metabolism likely acquires a high turnover status after birth comparable to that seen in post-menopausal osteoporosis patients.

**Table 2:** Biological parameters in post-partum fracture patients.

	Mean	Range
Age (years)	35.6 ± 4.5	26–46
BMI (kg/m^2^)	20.8 ± 3.1	16.1–32.8
total number of children post-partum	1.56 ± 0.71	1–3

Vitamin D also alters bone metabolism^25^; however, prior to birth, Vitamin D (25(OH)D) levels in the 79 subjects were lower than sufficient (20 ng/ml) and did not change significantly after partum (Fig. 1). However, levels of 1,25(OH)_2_D3_,_ an active form of vitamin D3, were significantly down-regulated after partum (Fig. 1). Vitamin D levels are also reportedly inversely correlated with PTH levels^14,25^. Indeed, we observed low 1,25(OH)_2_D3 and increased PTH after birth in our subjects (Fig. 1). Levels of the hormone, prolactin, which promotes breast milk secretion, significantly decreased at one month after partum (Fig. 1). Finally, levels of serum calcium (Ca), phosphorus (P) and creatinine (Cr) significantly increased after partum (Fig. 1).

### Post-partum women exhibit high bone turnover status

Next, we analyzed changes in bone metabolic status after childbirth in the group of 79 pregnant subjects (Fig. 2). Bone resorption markers such as serum tartrate resistance acid phosphatase 5b (TRACP5b), an osteoclast marker, and urinary deoxypyridinolin (uDPD), a type 1 collagen cross-linking protein, were both significantly elevated (Fig. 2a). However, other bone resorption markers including urinary type 1 collagen cross-linked N-telopeptides (uNTX) and urinary hydroxylproline, a collagen-specific amino acid released from bone matrix by osteoclast bone resorption, were significantly decreased in post-partum women (Fig. 2a).

Moreover, bone-forming parameters, namely bone alkaline phosphatase (BAP), a bone specific isoform, pro-collagen type 1 amino-terminal propeptide (P1NP), which is cleaved from pro-collagen and released into the circulation during bone formation, and osteocalcin and uncarboxylated osteocalcin (ucOC), both produced by osteoblasts, significantly increased after partum (Fig. 2b). Thus, although uNTX and hydroxylproline levels decreased, we conclude that overall bone metabolic status in these subjects reflected high turnover, comparable to that seen in post-menopausal osteoporosis patients.

### Other metabolic changes seen in women after partum

Hyperlipidemia is frequently seen in the patients with post-menopausal osteoporosis^26^. Since post-partum women exhibited metabolic changes similar to those seen in post-menopausal patients, we analyzed lipid status after birth in the group of 79 pregnant subjects. Unlike post-menopausal osteoporosis patients, however, total cholesterol (T-Cho), low- and high-density lipoprotein cholesterol (LDL and HDL), and triglycerides (TG) decreased significantly after partum (Fig. 3a). Moreover, levels of hemoglobin A1c (HbA1c), a glyco-metabolic marker, and homocystein, a methionine metabolite, decreased and increased, respectively, following birth in these subjects. (Fig. 3b). Levels of pentosidine, which marks advanced glycation end products (AGEs), and creatine kinase (CPK), an enzyme expressed in muscle and brain, were comparable before and after birth (Fig. 3b). Finally, aspartate transaminase (AST) and alanine transaminase (ALT), both liver enzymes, and albumin (Alb) increased significantly, while lactate dehydrogenase (LDH), which catalyzes lactate conversion to pyruvate, was unchanged before and after birth (Fig. 3c).

### Lactation promotes bone-resorption but not bone-formation

**Figure 4:**
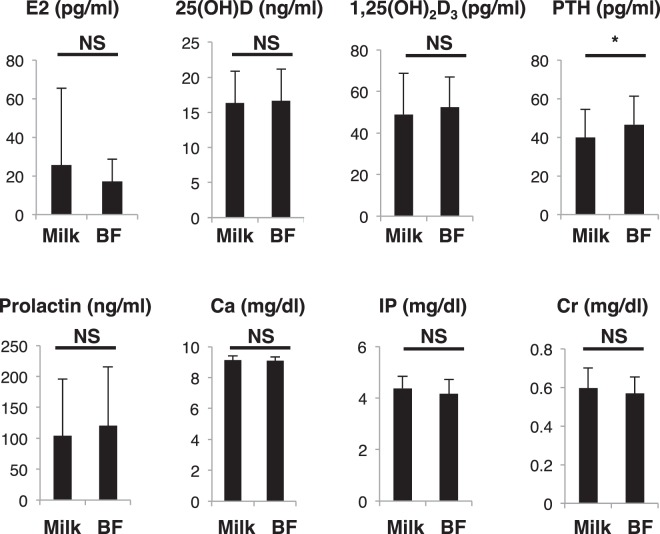
PTH levels significantly increase in breastfeeding-only cases. Sera were corrected from a group of 79 post-partum subjects, which were then subdivided into those who either breastfed only (BF) or fed their infants artificial milk or a mix of both (Milk) for one month after partum. Indicated parameters were analyzed and compared between groups. Data represent mean levels of indicated parameters ± SD (*n* = 34 for BF, *n* = 45 for Milk; *p < 0.05, NS not significant). E2, estradiol; PTH, parathyroid hormone; IP, inorganic phosphorus; Cr, creatinine.

**Figure 5:**
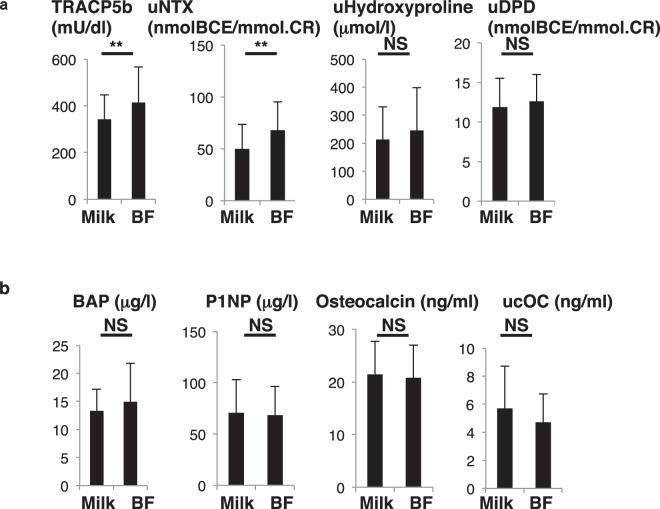
TRACP5b and uNTX levels significantly increase but bone-forming parameters are unchanged by lactation. Sera and urine were collected post-partum from 79 subjects who had been subdivided into two groups: those who either fed infants solely on breastmilk (BF) or those who fed their infants artificial milk or a mix of both (Milk) for one month after partum. Levels of indicated parameters were analyzed (**a**) bone-resorbing parameters; (**b**) bone-forming parameters) and compared between groups. Data represent mean levels of indicated parameters ± SD (*n* = 34 for BF, *n* = 45 for Milk; **p < 0.01, NS not significant). TRACP5b, tartrate resistant acid phosphatase 5b; uNTX, urinary type 1 collagen cross-linked N-telopeptide; uDPD, urinary deoxypyridinolin; BAP, bone alkaline phosphatase; P1NP, procollagen type 1 amino-terminal propeptide; ucOC, undercarboxylated osteocalcin.

**Figure 6:**
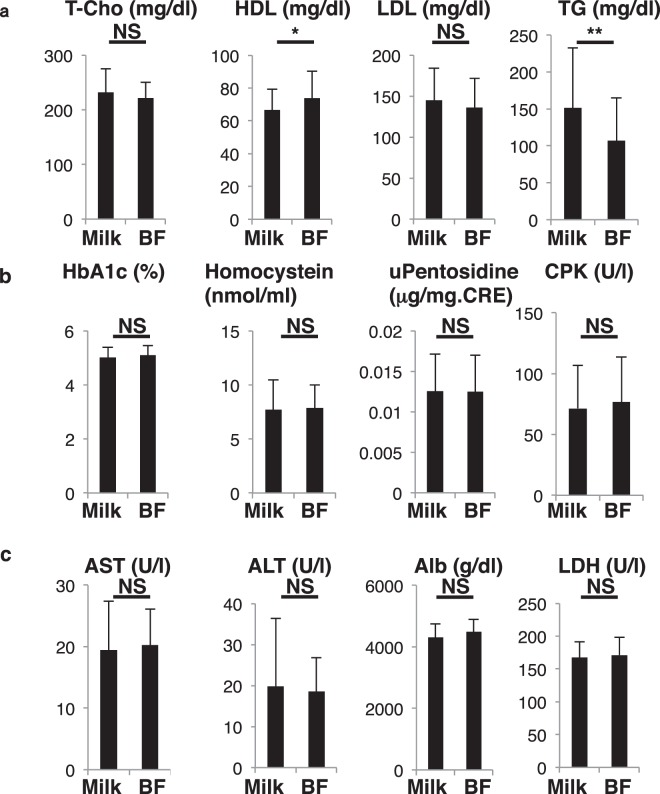
Most metabolic parameters are unchanged by lactation. Sera and urine were collected post-partum from 79 subjects, who were further subdivided into those who fed infants solely on breastmilk (BF) or those who fed infants artificial milk or a mix of both (Milk) for one month after partum. Samples were analyzed for levels of indicated parameters (**a**, lipid parameters; (**b**) glyco parameters; (**c**) liver parameters). Data represent mean levels of indicated parameters ± SD (*n* = 34 for BF, *n* = 45 for Milk; *p < 0.05, **p < 0.01, NS not significant). Pentosidine levels were analyzed in urine, and others were analyzed in sera. T-Cho, total cholesterol; HDL, high density cholesterol; LDL, low density cholesterol; TG, triglyceride; HbA1c, hemogloblin A1c; CPK, creatine phosphokinase; AST, aspartate aminotransferase; ALT, alanine aminotransferase; Alb, albumin; LDH, lactate dehydrogenase.

**Figure 7:**
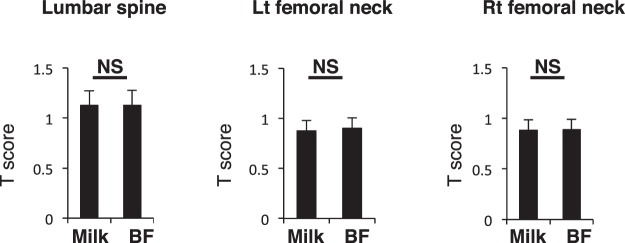
Lactation does not decrease BMD. Bone mineral density (BMD) as analyzed by a DEXA scan of lumbar spine (L2-4) and left and right femoral neck in 79 subjects subdivided into two groups: those who fed infants solely on breastmilk (BF) and those who fed infants artificial milk or a mix of both (Milk). Levels were compared at one month after partum between groups. Data represent mean levels of BMD ± SD (*n* = 34 for BF, *n* = 45 for Milk; NS not significant).

Lactation reportedly promotes calcium mobilization to infants and may promote decreased bone mass due to bone-resorption^1^. Among the 14 fracture subjects analyzed in our study, all fed their infants solely by breast milk. Among the 79 other subjects, 34 fed infants solely by breast milk, 4 by formula only, and the remaining 41 fed a mix of both. Therefore, we subdivided the group of 79 into two groups: a breastfeeding-only group (BF, *n* = 34) and a group comprised of mothers who fed their infants formula only or a mix of both (Milk, *n* = 45). There were no significant age or BMI differences between groups (Table 3). Between groups, only five parameters—PTH, TRACP5b, uNTX, TG and HDL levels—significantly differed among 41 analyzed (Figs 4–6). PTH is reportedly elevated during lactation to increase calcium levels^1^, thus, this result was anticipated. Coupled bone-resorption (marked by TRACP5b and uNTX) and bone-formation are regulated in parallel, and post-menopausal osteoporosis patients exhibit activation of both^18^. Increased TRACP5b and uNTX in the breastfeeding group likely represents stimulation of bone-resorption in this group; however, all bone-forming parameters were equivalent between groups Fig. 5), suggesting uncoupling of bone-resorption and bone-formation. HDL and TG significantly increased and decreased, respectively, but levels of other lipid, glyco and hepatic parameters were equivalent between groups (Fig. 6). Moreover, bone mineral density (BMD) in both the lumbar spine and femoral neck was comparable between groups (Fig. 7). BMD was not significantly inversely associated with either TRACP5b or uNTX after partum (Table 4). Furthermore, radiographic analysis revealed no evidence of vertebral fracture in any subject of the group of 79 at one month after partum (data not shown).

**Table 3:** Characteristics of breastfeeding and artificial milk/mixed groups.

	Breastfeeding	Artificial milk/mixed	*p* value
Age (years)	35.5 ± 4.1	35.7 ± 4.5	0.824
BMI (kg/m^2^)	21.0 ± 3.6	20.6 ± 2.7	0.631
total number of children post-partum	1.62 ± 0.70	1.51 ± 0.73	0.513

**Table 4:** BMD is not inversely associated with either TRACP5b or uNTX after partum.

	*β*	*p* value
BMD vs TRACP5b	0.156	0.092
BMD vs uNTX	−0.092	0.604

### Fracture subjects exhibit significantly low BMD

**Figure 8:**
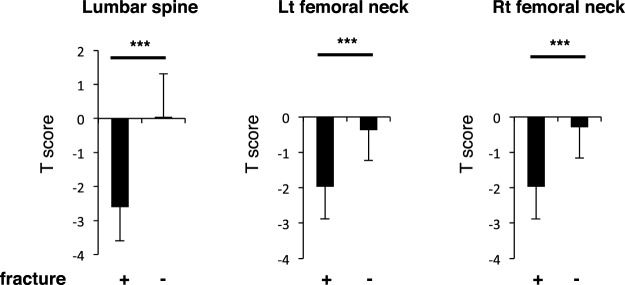
BMD is significantly lower in fracture versus non-fracture subjects under breastfeeding-only conditions. Bone mineral density (BMD) was analyzed by a DEXA scan in lumbar spine (L2-4) and left and right femoral neck in fracture (+) versus non-fracture (−) subjects at the first visit to our hospital or one month after partum, respectively. Levels were compared between groups. Data represent mean levels of BMD ± SD (*n* = 11 for fracture subjects, *n* = 34 for non-fracture subjects; ***p < 0.001).

Finally, we assessed potential causes of post-partum vertebral fractures in the group of 14 fracture subjects. Since these subjects fed their infants breast milk only, we compared various parameters between them and the larger group of 34 breastfeeding mothers in the larger group who did not show vertebral fractures. Age and BMI did not differ between groups (Table 5). Initially, we observed that BMD in lumbar spine and the femoral neck was significantly lower in fracture than non-fracture subjects (Fig. 8). We then analyzed metabolites and other factors in subjects’ serum and urine. Since fracture subjects #3, #5 and #10 had been treated with bisphosphonates (alendoronate and minodronate) before visiting our hospital, they were excluded from this study, leaving 11 subjects in the fracture group. We found that serum calcium and E2 were significantly higher in fracture versus non-fracture subjects, while PTH levels were significantly lower (Table 6). Phosphorus, albumin and creatinine levels were equivalent between groups (Table 6), and both groups exhibited comparable but low vitamin D status (25(OH)D levels <20 ng/ml) (Table 6). TG levels were comparable between fracture and non-fracture groups (Table 6).

**Table 5:** Characteristics of breastfeeding-only subjects with or without vertebral fractures.

	Fracture (+)	Fracture (−)	*p* value
Age (years)	35.1 ± 3.5	35.4 ± 4.1	0.783
BMI (kg/m^2^)	19.4 ± 2.2	21.0 ± 3.6	0.167
Number of injured vertebral bodies	3.7 ± 2.0	0 ± 0	<0.001
total number of children post-partum	1.18 ± 0.40	1.62 ± 0.70	0.056

**Table 6:** Biological parameters of breastfeeding-only subjects with or without vertebral fractures.

	Fracture (+)	Fracture (−)	*p* value
Calcium	9.8 ± 0.34	9.1 ± 0.23	<0.001
Phosphorus	4.4 ± 0.58	4.2 ± 0.56	0.213
Albumin	4.6 ± 0.31	4.5 ± 0.39	0.375
Creatinine	0.57 ± 0.09	0.57 ± 0.08	0.972
25(OH)D	18.8 ± 4.6	16.7 ± 4.5	0.392
PTH	29.6 ± 17.3	46.6 ± 14.7	0.009
E2	31.9 ± 24.2	17.2 ± 11.4	0.014
TG	108.9 ± 103.6	107.1 ± 58.3	0.944

**Figure 9:**
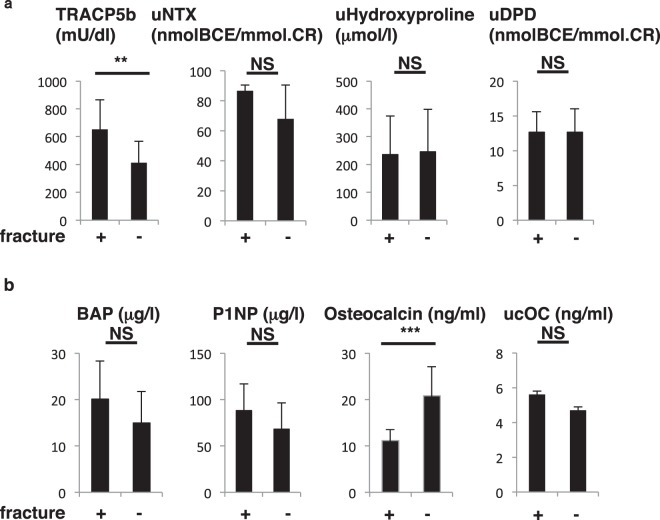
Fracture subjects show increased TRACP5b and decreased osteocalcin levels. Sera and urine were corrected from post-partum subjects with or without vertebral fractures, as described in Fig. 8. All subjects in both groups fed their infants breast milk exclusively. Levels of indicated parameters were analyzed. Data represent mean levels of indicated parameters ± SD (*n* = 11 for fracture subjects, *n* = 34 for non-fracture subjects; **p < 0.01, ***p < 0.001, NS not significant).

Analysis of levels of bone remodeling factors revealed that the fracture group exhibited significantly higher TRACP5b, a bone-resorption factor, and lower osteocalcin, a bone-forming parameter, than did the non-fracture group (Fig. 9). Levels of other bone-forming factors, namely ucOC, BAP and P1NP, as well as bone-resorption parameters, uNTX, uDPD and uHydroxyproline, were comparable between groups (Fig. 9).

## Discussion

Pregnancy and lactation induce significant metabolic changes in the maternal skeleton. Initially, intestinal calcium absorption is elevated by increased levels of active vitamin D3, 1,25(OH)_2_D_3_, and PTH. If this is insufficient to meet calcium demands, calcium is then mobilized from the maternal skeleton by either osteoclast activity or osteocyteic osteolysis mechanisms, which in turn, cause reduced bone mass and in some rare cases fragility fractures in mothers. Here, we evaluated 14 maternal fragility vertebral fracture cases marked by severe back pain within three months after partum; all of those subjects had solely breastfed their infants. We then enrolled 79 additional pregnant subjects, some who eventually breastfed their infants and some who did not, and analyzed metabolic changes before and after partum. In the latter group, after partum, E2 levels drastically decreased relative to those before partum, and differences in E2 levels occurring over these 3 months were approximately 100 times greater than those seen at menopause. Concomitantly, bone acquired a high turnover status, and bone-resorption and bone-formation were activated in a manner seen in post-menopausal osteoporosis patients. Among post-partum subjects in the group of 79, women who fed their infants solely by breastfeeding exhibited activities indicative of uncoupled bone homeostasis, namely high osteoclast activity without accompanying increased osteoblastic activity, relative to mothers who fed their infants only formula or a mix of both.

The other group of 14 subjects exhibiting fragility fractures showed significantly lower BMD than did non-fracture subjects, even those who had breastfed their infants. Low vitamin D levels are reportedly associated with fragility fractures in the elderly^27–29^. We also detected low vitamin D levels in our fragility fracture subjects (Table 6). Low vitamin D activity in young females could be due to either avoidance of sun exposure or limited food intake^25^. Vitamin D (25(OH)D) levels in mothers’ sera reportedly correlate positively with levels in sera of newborns^30^. Moreover, vitamin D levels in mothers’ breast milk have also been demonstrated to be positively correlated with those in mothers’ sera^31^, and our subjects exhibited low vitamin D status (Table 6). Based on all of these findings, we recommend monitoring vitamin D status of mothers and infants. Nonetheless, low vitamin D status levels were comparable between fracture and non-fracture subjects (Table 6).

Menopause is a well-known risk for osteoporosis in the elderly, and a transient menopausal state is normally seen in women after partum. Menopausal estrogen deficiency promotes osteoclast activation. Here, we observed ~100 times greater E2 levels in young subjects before partum than in normal pre-menopausal females, and these levels fell to those normally seen in post-menopausal female after partum in a very short period (Fig. 1). Estrogen maintains bone mass by acting as an osteoclast-inhibiting hormone, and thus, more significant estrogen loss seen after partum than that due to menopause may promote greater osteoclast activation, leading to bone loss. Indeed, we observed induction of bone status indicative of high turnover, with osteoclast activation after partum. The physiological consequences of significant E2 decreases after partum are unclear, but resultant osteoclast activation likely triggers calcium mobilization from maternal skeleton required for lactation, and this may cause bone loss and fragility fractures as well.

In fracture subjects, fragility fractures were only seen in vertebrae (Table 1). Indeed, BMD reduction was greater in lumbar spine than in the femur of fracture subjects (Fig. 8). But femoral BMD was also significantly lower in fracture than non-fracture subjects (Fig. 8), suggesting that subjects could be at risk for other fragility fractures such as hip fractures. Hip fractures most commonly occur due to falls in the elderly^32^. In the current study, subjects were younger and less likely to experience falls. Instead, mechanical loading of the maternal spine, possibly due to carrying or holding babies, could constitute a mechanical stress associated with vertebral fragility fractures. Osteoporosis prevalence, as diagnosed in the general population by low BMD in lumbar spine, is reportedly lower than that seen in the femoral neck in both males and females^33^. However, 8 of 11 fracture subjects analyzed exhibited lower BMD in lumbar spine than in the femoral neck (data not shown), suggesting that pregnancy and/or lactation decrease BMD in vertebral bones, which are rich in cancellous bone relative to femoral necks, which are cortical bone-rich areas. Thus osteoclasts likely tend to resorb bone more efficiently from cancellous than cortical bones.

Subjects who exclusively breastfed exhibited evidence of uncoupled bone-resorption and bone-formation (Fig. 5). At present, how maternal uncoupled bone status is regulated is unclear. Interestingly, among bone-resorption parameters, TRACP5b was elevated in subjects who showed fractures, but levels of uNTX, uDPD and uHydroxyproline, all products of collagen degradation accompanying bone-resorption, were not (Fig. 9), suggesting that collagens are not degraded during lactation. Thus, TRACP5b elevation mediated calcium resorption and mobilization calcium but apparently did not alter collagen in the maternal skeleton.

Our study has some limitations. Our subjects were not asked about nutritional status, including dietary vitamin D and calcium intake. We also lack BMD data relevant to fracture or non-fracture subjects prior to initiation of the study. Changes in bone metabolism seen after birth in breastfeeding subjects strongly suggest that high bone turnover followed by uncoupled bone-resorption and lower BMD is brought on by pregnancy and lactation, an idea supported by prospective studies^34–36^. However, fracture subjects exhibited significantly lower BMD than non-fracture subjects who had breastfed, suggesting that fracture subjects may have had low pre-existing BMD such as idiopathic osteoporosis before pregnancy and that their vertebral fractures were brought on by metabolic changes and mechanical stresses experienced during pregnancy and lactation. This conclusion is supported by our present two studies. Nonetheless, further studies are needed to validate our results.

**Figure 10:**
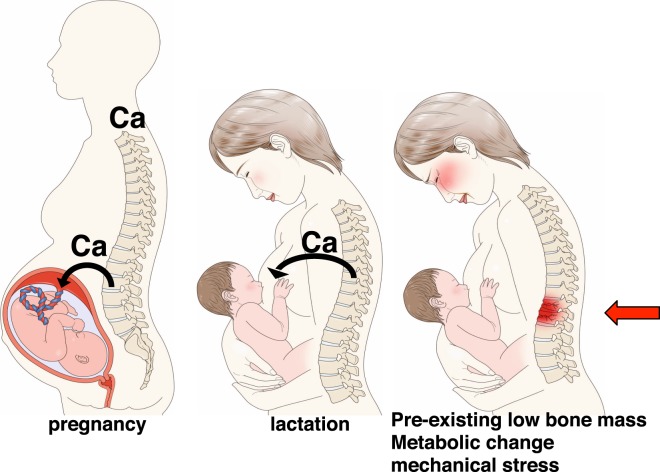
Schematic showing mechanisms underlying bone fragility or low-energy fractures due to pregnancy and lactation. Fragility or low-energy fractures can occur in the maternal skeleton during pregnancy and lactation via mechanisms shown. Fetal calcium is supplied from the maternal skeleton, promoting high bone turnover due to a drastic drop in estrogen levels after partum. These outcomes promote osteoclast activity without accompanying osteoblast activity and a loss in bone mass in breastfeeding-only conditions. Note that low bone mass may exist before pregnancy, and a combination of bone metabolic changes and mechanical stresses may also play a role in fragility or low-trauma fractures.

In summary, this paper shows overall that rare fragility fractures seen after partum may emerge from a combination of factors, among them, calcium mobilization from the maternal skeleton to the fetus, high bone turnover due to marked estrogen decreases after childbirth, lactation-dependent uncoupling of osteoclast from osteoblast activity, pre-existing low bone mass, or a combination of metabolic changes and mechanical stresses (Fig. 10). Thus, BMD examinations are recommended for young women of child-bearing age.

## Conclusions

In the current study, we assessed fourteen fragility vertebral fracture cases after partum. We also enrolled an additional 79 pregnant females, some who later breastfed and others who did not, and analyzed their serum and urinary parameters approximately one month before and after partum. In the latter group of 79, serum estradiol levels dramatically decreased after partum, and high bone turnover, as reflected by both bone-resorbing and -forming parameters, was seen relative to pre-partum conditions. However, none of the 79 subjects showed radiographic evidence of vertebral fractures after partum. By contrast, among the 14 fragility fracture cases, all of whom breastfed their infants, bone mineral density (BMD) was significantly lower than that seen in non-fracture, breastfeeding-only subjects in the group of 79. Bone-resorption increased but bone-formation did not in breastfeeding-only subjects compared with subjects who fed their infants formula, artificial milk, or a mix of the two. Taken together, this work shows that bones acquire high turnover status after partum, and that low BMD resulting from metabolic changes due to breastfeeding combined with mechanical stress likely causes fragility fractures after partum in a small subset of pregnant women. Thus, checking BMD before planning a pregnancy is recommended for women and this should be advised by physicians who are dealing with fracture patients, or obstetricians and gynecologists who are advising young female patients who are thinking of a pregnancy.
